# Psychological Well-Being and Intrinsic Motivation: Relationship in Students Who Begin University Studies at the School of Education in Ciudad Real

**DOI:** 10.3389/fpsyg.2020.02054

**Published:** 2020-09-10

**Authors:** Ángel Luis González Olivares, Óscar Navarro, Francisco Javier Sánchez-Verdejo, Álvaro Muelas

**Affiliations:** ^1^Faculty of Education, University of Castilla-La Mancha, Ciudad Real, Spain; ^2^Faculty of Education, University Center Villanueva, Complutense University of Madrid, Madrid, Spain

**Keywords:** young people, well-being, intrinsic motivation, vocational character, Degree of Pre-school and Primary Education

## Abstract

More and more studies and research have found a positive relationship between the participation of young peoplein altruistic activities and helping others. It is interesting to discover the relationship that personal and vocational satisfaction play in the preparation and training for the teaching profession. For students who begin university studies related to teaching, their psychological well-being and motivation toward this activity are very relevant aspects to consider. The access to and attainment of a university degree with a great vocational character, such as that of Pre-school Teacher or Primary Education Teacher, can benefit the students under their tutelage. An adequate motivation and a psychological well-being might favor future educational professionals’ personal balance and will impact their students. This research aims to analyze the degree of psychological well-being and its relationship with the motivation toward starting the teaching career. A sample of 233 students and students aged between 17 and 19 who are beginning university studies at the School of Education of Ciudad Real was selected. All participants were enrolled in the first year of the Degree in Pre-school and Primary Education. The relationship between the psychological well-being of the students and their motivation toward attaining a professional teaching career was analyzed. Other variables, such as age or gender were also taken into account. A quantitative study was carried out and the Ryff Psychological Well-Being Scale (RPWS) and the validated Intrinsic Motivation Questionnaire (IMI) were used. Once the results were analyzed, it was observed that there is a positive correlation between the perception of psychological well-being that the participants have and their motivation toward the beginning of university studies. The focal point of these future teachers is direct teaching with the students of the early stages of Pre-school and Primary Education. There are also some significant differences, considering the age and gender of the participants. The vocational character of university preparation for the teaching profession may determine that students who begin their degree studies have an important motivation toward the performance of their professional future.

## Introduction

Human well-being has been the current objective of research for decades. In order to know what factors help to achieve human well-being, one first must prioritize the concept of well-being and what the factors are that help to achieve well-being. Human well-being studies and research have focused on two practices that have traditionally approached the concept from two hegemonic points of view. On the one hand, with the Hedonic current of well-being, the focus is centered on a construct of subjective well-being. And on the other, with the Eudaimonic current, the focus is based on a construct of psychological well-being.

With many concepts and approaches in common, subjective well-being and psychological well-being are two related constructs, but conceptually different (Keyes et al., 2002; Linley et al., 2009). For Barra et al. (2013), subjective well-being is concentrated on vital satisfaction and happiness, considered as something with lasting positive affects over negative ones, and psychological well-being is channeled into more transcendental aspects in a person’s life. Along the same lines, Ryan and Deci (2001), the Hedonic approach (subjective well-being) focuses on happiness and defines well-being in terms of achievement and avoidance of pain; and the Eudaimonic approach (psychological well-being) focuses on meaning and self-realization, defining well-being in terms of the degree to which a person is fully functioning.

Psychological well-being is characterized by subjectivity, the presence of positive indicators and the absence of negative factors, such as a global assessment of life (Diener, 1984). In this sense, psychological well-being itself is not only a reflection of having a happy life (Cuadra and Florenzano, 2003), but also of having the ability to overcome complicated, painful, and conflicting processes. Therefore, it includes both elements related to the affective and evaluative fields of analysis and reflection (Vázquez and Hervás, 2008).

Ryff (1989) outlined the “Multidimensional Model of Psychological Wellbeing,” also called “Model of Constructive Multidimensionality.” Already in 1985, Ryff and Keyes proposed a descriptive model of psychological well-being consisting of six categories: Acceptance of oneself, Positive Relationships with other people, Autonomy, Mastery of the environment, Purpose in life, and Personal Growth. The acceptance of oneself is the positive consideration toward oneself, being aware of the limitations. People learn to accept themselves because we admit how we are to the situations and circumstances we experience, but we also consent because the experience has given us the opportunity to value, appreciate, and ignore the scenarios to which we have been exposed. In the same way, our action can also be of submission, because one is not always right. In this sense, recognizing and agreeing are not an act of ignominy, but of confessing not to have learned correctly or not enough. In itself, accepting oneself is an important fact of knowledge, because for oneself to know oneself is to know oneself correctly and objectively. The category of Positive Relationships with other people involves being able to carry out constant relationships with others, developing trust, and affection. Socializing is learning socially because “el ser humano está dotado de sociabilidad, que es la disposición de la persona a estar en sociedad”^1^ (Quicios et al. 2019, p. 57). Just as life progresses continuously, the relationship with society is a permanent process in time. The surprise is the result of the interaction among people. Being the correspondence between tangible individuals, it enriches cognitively, it grows emotionally and perfectly shapes behavior. The third category can be explained with the ability to emancipate, properly manage interests and priorities, in addition to controlling behavior itself. The autonomy of the will is the faculty and right of the people to establish norms of conduct for oneself and one’s relations with others taking into account ethics. The Domain of the Environment, considered as the surrounding environment, reflects the ability of the person to interact, adapt, and influence it according to personal needs, interests, and desires. It is the ability of the individual to choose and create environments conducive to meet their own needs and develop optimally (Ryff, 1989), becoming one of the greatest determinants of human psychological well-being (Ryan and Deci, 2001). The category of Purpose in life is specified in the definition of attainable goals that symbolize the importance of past and future experiences. These past experiences are the result of lived existence; felt, perceived, or experienced within us, as well as the interaction with the environment, context, and society. In relation to subsequent practices, the starting point is the experiential identity created, projected with the interest and hope of improving, correcting, or discovering successful world-studies. The last category, called Personal Growth, is the strategies and skills that strengthen and optimize one’s own abilities and potentials, in itself the itinerary of growth and maturity of the person. It is each one’s fate that helps one to grow, to feel the desire for the perception of one’s own path, and to appreciate and enjoy the good and all that fullness entails.

In addition to these categories, there are variables such as age, gender, and culture that influence psychological well-being (Rangel and Alonso, 2010). And Eudaimonic (psychological) well-being would involve acting in a constructive, socially beneficial way and would be conducive to Personal Growth (Deci and Ryan, 2008; Wood et al., 2009). In this case, an instrument has been used that assesses the psychological well-being of participants. It is composed of six dimensions, plus the sum of all of them. It is the Ryff Psychological Well-Being Scale (RPWS).

Many relevant studies have detailed that subjective well-being has a strong analogy with personality (Diener and Lucas, 1997). In this investigation, we focus on an important aspect of personality: motivation. This concept has a subjective character since it is a feeling or personal state whose origin can be diverse. Chekola (1974) states that there is a direct relationship between the degree of motivation that an individual can feel and the desire(s) that led him to be motivated.

Throughout the first half of the twentieth century, the mechanistic paradigm prevailed when describing motivation theoretically, where variables such as activation, instinct, or impulse were considered (Hull, 1925; Tolman, 1932). But this concept was changing in the face of the appearance of the idea of reward in relation to motivation. The emerging cognitive paradigm in the second half of the last century establishes that a wide variety of rewards can involve different motivations, which were gradually imposed on the mechanistic approach. This new perspective focuses on the so-called motivation of achievement, which is considered the motivation driven toward the achievement of goals. The first author who established this concept was Atkinson (1964), although the contribution of Heider (1958) to this theory and Jones and Davis (1965), Kelley (1967), or Weiner (1985) can also be highlighted. All these contributions established the basis for the different expectations-value theories that understand motivation as a product of expectations in relation to the expected result.

Maslow (1954) considered that desires led to need or the belief of need. This led him to develop his “Motivation Theory,” which establishes a hierarchy of needs, in order of importance for all human beings. These needs can go from the basic needs which, once they have been satisfied, lead us to a higher level. He classifies the needs into five types and they would go in ascending order. From the theory of Maslow arises the approach of McClelland (1958), the “Theory of Need for Achievement.” For this author, the motivation would be determined by three types of needs: need for achievement, need for power, and need for affiliation. These three types of needs are very present in people. Unlike Maslow’s proposal where desires led to needs, the needs of McClelland are learned. However, neither author addresses the relationship among the maturity of the individual, their needs, and motivation. It must be taken into account that the needs may vary throughout life, correspondingly evolving our motivations.

There is currently no unanimity in terms of the concept of motivation, since different views coexist depending on the objectives of achievement:

- Atkinson (1964) related the expectations of achieving the goals with the competencies and data that come from other people.
- Motivation has its origin in the way in which each one feels personally (how he does it, if he does it himself or what leads him to do it), what he intends to achieve (incentives), the intrinsic or extrinsic motivation, or what one can contribute to society (recognition; Maehr and Braskamp, 1986).
- Our own concept of self-efficacy influences motivation from the point of view of Social-Cognitive Learning theory (Bandura, 1986). Our learning will be conditioned on our good work and expectations.
- In reference to causal attributional theories (Dweck and Leggett, 1988), depending on the relationship between personality and the environment behavioral goals will be determined.

Authors such as Manassero and Vásquez (2000) make a thoroughly study of the evolution of the concept of motivation, highlighting the motivation for achievement, the instruments that can measure it and the evaluation of its categories. But it is also considered necessary to know the nature of the motivation to be able to determine whether human behavior depends on it and, if so, to see why it is. Authors such as Deci and Ryan (1985, 1991) developed the Theory of Self-Determination, in relation to motivation (Deci et al., 1991; Deci, 1992; Ryan and Deci, 2000). According to these authors, the fact that being motivated can encourage the individual to do something or persist in a certain behavior, in a specific context (Ryan and Deci, 2000). They determine that there are two types of motivation: intrinsic and extrinsic. The intrinsic would be the inherent tendency to look for novelties and challenges, expand and exercise the ability to explore and learn. On the other hand, extrinsic motivation refers to the performance of an activity to achieve some independent result and, therefore, contrasts with intrinsic motivation, which refers to performing an activity for the inherent satisfaction of the activity itself. In addition, some needs are established that are considered psychological and innate (competence, autonomy, and relationship) and also motivate the individual to initiate a certain behavior.

Going deeper into intrinsic motivation, Grant and Dweck (2003) consider that goal achievement has a positive influence, both on intrinsic motivation and performance when an individual faces prolonged challenges. Pintrich and de Groot (1990) establish three components to study motivation: personally perceived competence to get involved in the task, beliefs about the benefits of a specific task, and affective-emotional reactions. On the other hand, McInerney et al. (2003) believe that a previous goal setting has intrinsic motivation that directs energy toward achieving the desired results.

Intrinsic motivation is a resource that teachers have traditionally used, not only as a natural source of learning, but also to achieve the benefits raised from the needs. The pursuit of certain activities is sought for the inherent satisfaction of the participant, rather than for the consequence. The participant is sought to act more for fun or challenge than for external rewards or pressures. Depending on the fulfillment of basic psychological needs, intrinsic motivation can increase and has an active position in the performance of any activity (Ryan and Stiller, 1991; Ryan and Deci, 2000).

In the recent years, many similar researches have been realized, with a wide variety of instruments (Sekhar et al., 2013; Tamilmani et al., 2019). In this case, Intrinsic Motivation Inventory (IMI) was used to know the participants’ motivation. This test has been used in many investigations at different times, from people of different ages (Eskeles, 1982; Mendez et al., 2018; Heilat and Seifert, 2019), or even professors as in the present investigation (Demir, 2011; Liu et al., 2019). Teachers may be exposed to external and internal motivation. It is important that motivation is intrinsic, so that training and preparation for future teaching work translate into a better teaching-learning process.

## Materials and Methods

### Objectives

The purpose of this paper is to show the state of well-being of students who begin university studies for a teaching degree, both in early childhood and Primary Education. The motivation of the participants toward the beginning of a career of a vocational nature such as teaching is also analyzed. The specific objectives that are more concrete are the following:

- To know the well-being status of future teachers when they begin their studies.
- To know the motivation toward the beginning of a teaching career.
- To check the relationship between the well-being state and intrinsic motivation.
- To check whether there are differences in the well-being state or motivation depending on their university studies or gender.

### Participants

**Figure 1:**
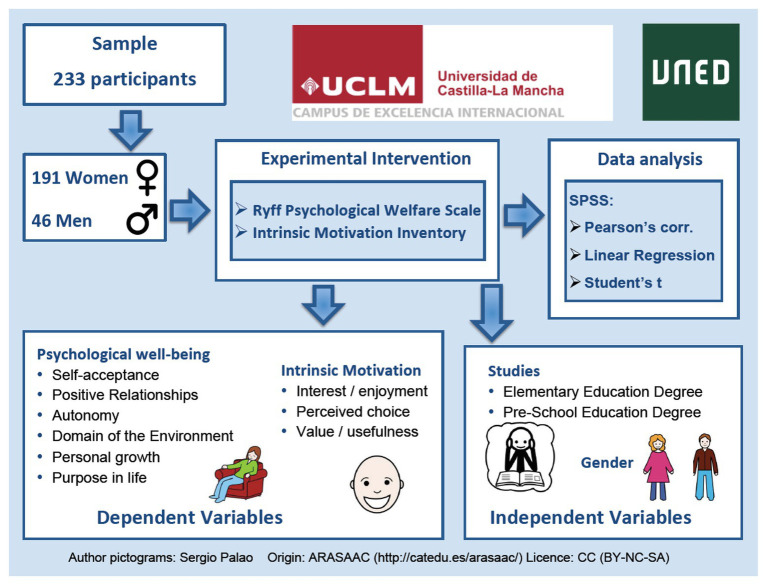
Research outline.

Figure 1 shows the research design of the present study. A sample of 233 university students, of both genders (women *n* = 191 and men *n* = 46) was used; with an average age of 18.71 years, and similar socioeconomic level, who are in the first year of a Master’s Degree in Primary and/or Pre-school Education Teaching at the School of Education of the University of Castilla-La Mancha in Ciudad Real. It should be noted that, in general, there is a greater number of females than males studying for a teaching degree.

### Tools Used

Participants had to fill in a validated 64-item questionnaire, corresponding to the RPWS and the IMI. Enough time was available for all students to complete these questionnaires. In almost all cases, the duration was between 10 and 15 min.

The genders of the participants and the studies have been taken into account, either a degree in Early Childhood Education or a degree in elementary education. On the other hand, regarding the dependent variables, the dimensions of the two validated questionnaires discussed above have been taken into account.

The RPWS is an instrument that has a total of six scales and 39 items to which participants respond using a response format with scores between 1 (strongly disagree) and 6 (strongly agree). The Spanish version was created by Díaz et al. (2006) adding an adequate consistency level (Cronbach’s *α* of 0.70). In addition, the scales showed an excellent level of adjustment to the theoretical model proposed by van Dierendonck. In the university population, Véliz-Burgos (2012) obtained acceptable psychometric properties.

The variables derived from this questionnaire are:

- Self-acceptance: Everyone wants to feel good about themselves, even recognizing their limitations.
- Positive Relationships: The ability to maintain positive relationships with other people. People need to maintain stable social relationships and have friends that they can trust.
- Autonomy: For Ryff and Keyes (1995) people need to establish themselves in their own convictions (self-determination) and maintain their independence and personal authority in order to sustain their own individuality in different social contexts.
- Domain of the Environment: The personal ability to choose or create favorable environments to satisfy one’s desires and needs.
- Personal Growth: Optimal positive functioning not only requires the characteristics mentioned above, but also needs constancy to develop one’s potential, to continue growing as a person and maximizes their abilities. For Keyes et al. (2002) it is the category called Personal Growth.
- Purpose in life: People need to set goals, define a series of objectives that allow them to give their lives a certain meaning. They need a purpose in life.

On the other hand, the IMI has been used. This instrument performs a multidimensional measurement to evaluate the subjective experience of the participants in relation to the motivation toward a specific activity (Deci and Ryan, 1985). Different versions of this questionnaire can be found. In this case, a version that includes three categories has been chosen. This version has been considered to be the most suitable for people of this age. The validated questionnaire consists of 25 items, which are answered with scores between 1 and 7, depending on the perception of motivation by the participant. In this case, to fill out the questionnaire was enough with about 10 min. This test has been validated in several studies, confirming that it provides adequate reliability and validity for a study like this (Kooiman et al., 2015; Ostrow and Heffernan, 2018). The variables that are derived from this questionnaire are:

- Interest/Enjoyment is considered a measure of the intrinsic motivation of oneself, depending on the interest aroused by the activity.
- Perceived Choice is a positive predictor of self-reporting and measures of intrinsic motivational behavior depending on the participants’ choice in relation to the proposed activity.
- Value/Usefulness subscale is used in internalization studies, the idea is that people self-reflect and self-regulate with respect to the activities they experience as useful or valuable for themselves.

The analysis of the data also included the general values of the variables of both questionnaires. On the one hand, an overall value of the well-being state, obtained from the sum of the six variables showed previously (General Psychological Well-Being). On the other hand, the sum of the three intrinsic motivation variables provides a total and unified value of the used instrument (General Intrinsic Motivation).

### Hypothesis and Analysis

Based on the questionnaires and taking into account the exposed variables, the following hypotheses were established:

- *H_1_*: There is a correlation among the subscales of the RPWS.
- *H_2_*: There is a correlation among the subscales of the IMI.
- *H_3_*: There is a correlation among the subscales of the RPWS and the IMI.
- *H_4_*: There are differences in the psychological well-being of the participants according to gender.
- *H_5_*: There are differences in the psychological well-being of the participants depending on their course of studies.
- *H_6_*: There are differences in the intrinsic motivation of the participants according to gender.
- *H_7_*: There are differences in the intrinsic motivation of the participants depending on their course of studies.

The results of the two validated questionnaires applied were exported to the Statistical Package for the Social Sciences (SPSS) software with the intention of performing the corresponding data processing. Three statistics have been selected that were the most suitable for the present investigation. Pearson’s correlation allows one to distinguish if there is any correspondence among the different variables. Linear regression analysis has also been performed to analyze the relative influence of the different intrinsic motivation factors on psychological well-being. Finally, note that the contrast of means through Student’s *t* has been used. This test allows verification as to whether there is a significant difference among the average values obtained from the independent variables, the gender of the participants and their course of studies, the Elementary Education Degree, and the Pre-school Education Degree.

## Results

Based on the answers provided by the students participating in this research, the relationship or dependence among the different selected variables has been analyzed. First, the correlation among the categories of both questionnaires is presented separately, and thus verifies that these categories are related to each other. Subsequently, these variables are contrasted, first by Pearson’s correlation and later with the linear regression test. Finally, an analysis indicates if all these categories show some difference depending on gender or the students’ degree program.

Table 1 shows that there is a very high correspondence in all categories of the IMI questionnaire. In all cases it correlates significantly at the level of 0.01, in some cases with values that are quite close to one. These results confirm the coherence among the three categories that make up this instrument and that a high value in one of them implies a similar result in the others. It is an expected result that allows us to confirm this trend.

**Table 1:** Correlation (Pearson) among the intrinsic motivation categories.

		Interest/Enjoyment	Perceived Choice	Value/Usefulness	General Intrinsic Motivation
Interest/Enjoyment	Corr. Pearson	1	0.713^**^	0.604^**^	0.901^**^
	Sig. (bilateral)		**0.000**	**0.000**	**0.000**
Perceived Choice	Corr. Pearson		1	0.473^**^	0.839^**^
	Sig. (bilateral)			**0.000**	**0.000**
Value/Usefulness	Corr. Pearson			1	0.824^**^
	Sig. (bilateral)				**0.000**
General Intrinsic Motivation	Corr. Pearson				1
	Sig. (bilateral)				

** The correlation is significant at the level 0.01 (bilateral).

As in the previous case, a very high correspondence can be found among all the categories of the Ryff questionnaire. In Table 2, it can be seen that they are also all significant values at the 0.01 level. Therefore, it can be affirmed that the well-being state perceived by the participants is reflected in all its categories: Self-acceptance, Positive Relationships, Autonomy, Environment Domain, Personal Growth, and Purpose in life. The same happens when the General Psychological Well-Being variable is taken into account, which combines a full value of all its categories.

**Table 2:** Correlation (Pearson) among the psychological well-being categories.

		Self-acceptance	Positive Relationships	Autonomy	Environment Domain	Personal Growth	Purpose in life	General Ps. Well-Being
Self-acceptance	Corr.	1	0.473^**^	0.506^**^	0.662^**^	0.361^**^	0.685^**^	0.822^**^
	Sig.		**0.000**	**0.000**	**0.000**	**0.000**	**0.000**	**0.000**
Positive Relationships	Corr.		1	0.376^**^	0.390^**^	0.553^**^	0.320^**^	0.721^**^
	Sig.			**0.000**	**0.000**	**0.000**	**0.000**	**0.000**
Autonomy	Corr.			1	0.437^**^	0.487^**^	0.446^**^	0.721^**^
	Sig.				**0.000**	**0.000**	**0.000**	**0.000**
Environment Domain	Corr.				1	0.253^**^	0.707^**^	0.753^**^
	Sig.					**0.000**	**0.000**	**0.000**
Personal Growth	Corr.					1	0.308^**^	0.681^**^
	Sig.						**0.000**	**0.000**
Purpose in life	Corr.						1	0.762^**^
	Sig.							**0.000**
General Ps. Well-Being	Corr.							1
	Sig.							

** The correlation is significant at level 0.01 (bilateral).

Table 3 shows the correspondence among the categories of the two questionnaires used. In general, it can be seen that there is a high correlation among most of the variables. In four of the categories of the Ryff questionnaire (Self-acceptance, Environment Domain, Personal Growth, and Purpose in life), a positive correlation can be seen with all the categories of motivation, including General Intrinsic Motivation. In addition, the correlation is very high, significant at level 0.01. The same happens in the case of the value of General Psychological Well-Being, correlating at a very high level with all the categories of intrinsic motivation. This trend is similar in the Autonomy category. It correlates with all the motivational variables, in almost all cases with a high level (*p* ≤ 0.01) except in the Perception Choice category, where it correlates at the level of 0.05. Finally, the category of Ryff’s Positive Relations questionnaire does not show a clear correspondence with motivation. It only correlates at level 0.05 with the category of Interest/Enjoyment.

**Table 3:** Correlation (Pearson) among psychological well-being and intrinsic motivation categories.

		Self-acceptance	Positive Relationships	Autonomy	Environment Domain	Personal Growth	Purpose in life	General Ps. Well-Being
Interest/Enjoyment	Corr.	0.331^**^	0.151^*^	0.200^**^	0.436^**^	0.328^**^	0.489^**^	0.426^**^
	Sig.	**0.000**	0.021	**0.002**	**0.000**	**0.000**	**0.000**	**0.000**
Perceived Choice	Corr.	0.272^**^	0.086	0.160^*^	0.383^**^	0.234^**^	0.412^**^	0.338^**^
	Sig.	**0.000**	0.191	0.014	**0.000**	**0.000**	**0.000**	**0.000**
Value/Usefulness	Corr.	0.260^**^	0.088	0.204^**^	0.405^**^	0.190^**^	0.379^**^	0.331^**^
	Sig.	**0.000**	0.183	**0.002**	**0.000**	**0.004**	**0.000**	**0.000**
General Intr. Motivation	Corr.	0.337^**^	0.127	0.221^**^	0.478^**^	0.292^**^	0.498^**^	0.427^**^
	Sig.	**0.000**	0.054	**0.001**	**0.000**	**0.000**	**0.000**	**0.000**

** The correlation is significant at level 0.01 (bilateral).

* The correlation is significant at level 0.05 (bilateral).

To analyze the relative influence of the different intrinsic motivation factors on psychological well-being, a linear regression analysis was performed. In Table 4, it can be seen that the motivational category that has the greatest influence on General Psychological Well-Being is Interest/Enjoyment. It is the only one of these three factors that has a significant beta value (*β* = 0.315) at 95%. On the contrary, the other two categories would have no significant influence on psychological well-being. Therefore, the linear regression indicates that the student’s Interest/Enjoyment, regardless of the Perceive Choice and the Value/Usefulness, is strongly related to psychological well-being.

**Table 4:** Regression coefficients. Dependent variable: General Psychological Well-Being.

	Standard coefficient			Correlations	
	B	*t*	Sig.	Zero order	Partial
Interest/Enjoyment	0.315	3.351	0.001	0.426	0.216
Perceived Choice	0.060	0.707	0.480	0.338	0.047
Value/Usefulness	0.113	1.510	0.132	0.332	0.099

The influence of the dimensions of psychological well-being on the different intrinsic motivation factors was also calculated. However, no significant values were obtained.

Next, we will analyze the differences in the different categories of the two questionnaires used according to gender and the studies in which the participants are enrolled, either the Degree in Pre-school or Primary Education.

**Figure 2:**
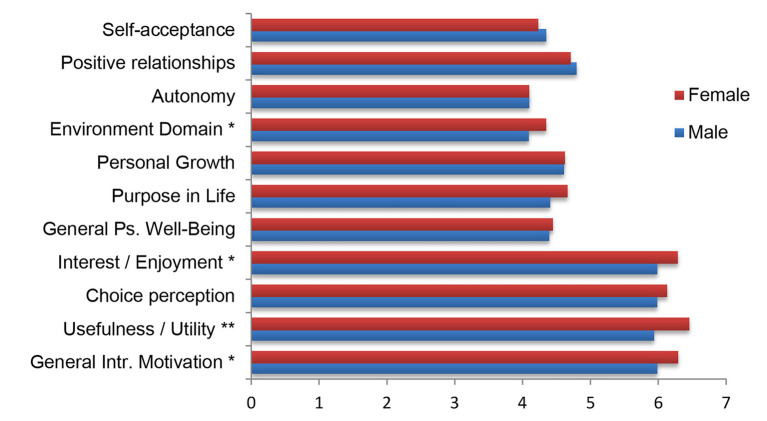
Difference of averages between psychological well-being and intrinsic motivation categories according to gender. ^**^The correlation is significant at level 0.01 (bilateral). ^*^The correlation is significant at level 0.05 (bilateral).

Figure 2 shows the differences among the means of the different categories in the two questionnaires administered. On the one hand, the Ryff questionnaire on psychological well-being shows a great equality in the values of almost all categories depending on the gender of the participants. Small differences are seen but mainly in the Domain of the Environment. This is the only case in which it can be affirmed that there are significant differences, since a *t* value of −1,970 (*p* ≤ 0.05) is obtained. Therefore, the female participants present a greater dominion of the environment.

There are no significant differences in the other psychological well-being variables (Self-acceptance, Positive Relationships, Autonomy, Personal Growth, and Purpose in life).

Regarding the other test used, the IMI questionnaire, there are more differences than in the previous case. It can be seen that female participants have a greater intrinsic motivation. On the one hand, differences in the values of the categories of Interest/Enjoyment and Value/Usefulness can be seen, as well as in the General Intrinsic Motivation taking into account the three categories of the questionnaire. In one of the cases, a very high difference can be seen with a level of significance at level 0.01 (Value/Usefulness). Specifically, a value of *t* = −2.693 (*p* = 0.01) is obtained. In the other two cases, the level of significance is lower of 0.05. Values of −2,490 (*p* = 0.013) and −2,429 (*p* = 0.019) are obtained, respectively, for the Value/Usefulness and the General Intrinsic Motivation. Finally, the Perceived Choice dimension has not presented significant differences and, therefore, the result is considered similar for the gender male and female.

**Figure 3:**
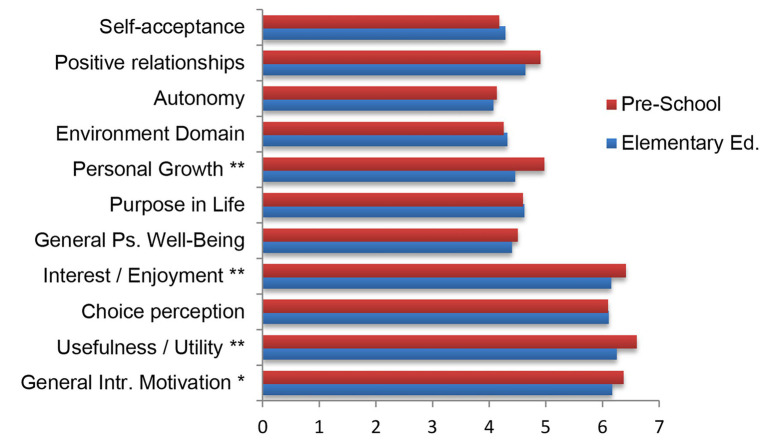
Difference of averages between psychological well-being and intrinsic motivation categories according to studies (Teacher Degree Elementary Education or Pre-school). ^**^The correlation is significant at level 0.01 (bilateral). ^*^The correlation is significant at level 0.05 (bilateral).

Regarding the degree program in which participants are enrolled, a trend similar to the previous case can be observed (Figure 3). In the categories that indicate the psychological well-being of the participants, there are hardly any differences. There is great equality except in some cases. Mainly, Personal Growth stands out, which is higher for students of the Master’s Degree in Early Childhood Education with a high level of significance (*p* ≤ 0.01). Values of *t* = −4.739 are obtained (*p* ≤ 0.001).

Regarding the categories of the IMI questionnaire, equality is observed in the case of the Perception Choice, as was the case in the previous case. Differences are seen in the other three. In the enjoyment interest and utility value, values of −2.678 (*p* = 0.008) and −4.020 (p ≤ 0.001) are obtained. In both cases, the level of significance is very high of 0.01. Finally, it is worth noting that there are also significant differences in the case of General Intrinsic Motivation. A value of −2.505 is obtained (*p* = 0.013), although in this case the level of significance is not high as in the other two variables (*p* ≤ 0.05).

## Discussion and Conclusions

Taking into account the general objective of this research, where it is intended to analyze the degree of psychological well-being and its relationship with to the motivation toward starting the a teaching career, it has been proven that there is a fairly high positive correlation between the perception of psychological well-being that the participants have and their motivation toward the beginning of university studies, whose professional focus is mainly direct teaching with the student of in the early stages of Infant Pre-school and Primary Elementary Education. Following the line of previous research (Keyes et al., 2002; Linley et al., 2009) and taking into account the first hypothesis (H_1_) raised in this research, there is a significant correlation among the variables analyzed in Ryff’s psychological well-being scale (Self-acceptance-Positive Relationships-Autonomy-Domain of the Environment-Personal Growth-Purpose in life-General Psychological Well-Being), in all cases and with a very clear correspondence. In relation to the second hypothesis of this research (H_2_), it can be confirmed because all the categories analyzed in relation to intrinsic motivation (Interest/Enjoyment-Perceived Choice-Value/Usefulness-General Intrinsic Motivation) correlate significantly.

In the third hypothesis (H_3_) raised, a significant correlation is observed, to a greater or lesser extent, among the variables analyzed in relation to psychological well-being and those of intrinsic motivation. Virtually all dimensions of well-being correlate positively with motivational dimensions.

This means that the greater the motivation of the participants takes place the better psychological well-being and vice versa. Only one exception is seen in the category of Positive Relationships with motivation. But of the three motivating factors, Interest/Enjoyment has a greater influence on Positive Relationships. This same trend is found in previous studies, such as those by Chekola (1974) or Diener et al. (1999). Therefore, the hypothesis H_3_ is almost entirely accepted and it is possible to affirm that there is a correlation between the subscales of the RPWS and the IMI.

Taking into account the psychological well-being of the participants of this study according to gender (Rangel and Alonso, 2010), and responding to the fourth hypothesis presented (H_4_), no significant differences in the results of the analyzed variables are observed, except in the Domain of the Environment category, in favor of the women. Therefore, the gender of the participants is not significant, and the results are virtually similar among men and women. Hypothesis H4 is rejected and it is possible to affirm that there are not any differences in the psychological well-being of the participants according to gender.

Following the same trend, there are hardly any differences in the psychological well-being of the participants based on the studies, except in the variable of Personal Growth, where the students of. Degree in Pre-school Education believes that their studies allow them to improve personally and professionally more than those enrolled in a Primary Education degree program. Therefore, hypothesis H_5_ is almost entirely rejected, which affirms that there are differences in the psychological well-being of the participants depending on their course of studies.

In relation to the intrinsic motivation presented by the students of the sample according to gender, and responding to the sixth hypothesis (H_6_) raised, one can see that female participants have a greater intrinsic motivation, mainly in the results of the categories of Interest/Enjoyment, Value/Usefulness, and General Intrinsic Motivation. Therefore, it is possible to accept the hypothesis H_6_, which affirms that there are differences in the intrinsic motivation of the participants according to gender.

Regarding the differences taking into account the course of studies of the participants, the trend is very similar. There are differences in the variables Interest/Enjoyment, Value/Usefulness, and General Intrinsic Motivation. Therefore, in all categories except the variable Perceived Choice, where similar samples are considered, the seventh study hypothesis is verified (H_7_, which affirms that there are differences in the intrinsic motivation of the participants depending on their course of studies).

In short, it can be concluded that the students of the Teacher Degree enrolled in a Master’s Degree Program (both Infant and Primary Education Pre-school and Elementary Education) who have a sense of psychological well-being, also have a great motivation of extrinsic character toward their formation in a profession as vocational as that of a teacher. The interest and/or enjoyment toward this work are the category that has the greatest influence on this well-being state. Regarding gender differences and studies, there is a greater motivation on the part of female students who are enrolled in Early Childhood Education.

## Future Research

As future lines of research, a study could be considered in different universities in other cities, where the perception of psychological well-being or motivation may be different from this study. It is also possible that different values are obtained when the studies carried out by the participants course of study of the participants does not have the same character as the Master Degree, a Master’s Degree in Education, which is focused on a very specific activity, teaching.

## Data Availability Statement

The datasets generated for this study are available on request to the corresponding author.

## Ethics Statement

Ethical review and approval was not required for the study on human participants in accordance with the local legislation and institutional requirements. The patients/participants provided their written informed consent to participate in this study.

## Author Contributions

All authors agree with the criteria and requirements of the publication. All authors contributed to the article and approved the submitted version.

### Conflicts of Interest

The authors declare that the research was performed in the absence of commercial or financial relationships that could be interpreted as potential conflicts of interest.

## Footnotes

^1^“The human being is endowed with sociability, which is the willingness of the person to be in society” (the translation provided is ours).
